# Moderating effect of cultural differences on the association between social media use and mental health outcomes in adolescents: A cross-cultural comparison study

**DOI:** 10.1371/journal.pone.0316365

**Published:** 2024-12-31

**Authors:** Betul Keles Gordesli, Mary Leamy, Trevor Murrells, Annmarie Grealish

**Affiliations:** 1 Florence Nightingale Faculty of Nursing, Midwifery & Palliative Care, King’s College London, London, United Kingdom; 2 Department of Mental Health and Psychiatric Nursing, İzmir Katip Celebi University, İzmir, Republic of Türkiye; 3 Department of Nursing and Midwifery, Health Research Institute, University of Limerick, Limerick, Ireland; Teikyo University - Hachioji Campus: Teikyo Daigaku - Hachioji Campus, JAPAN

## Abstract

The influence of social media on the mental health of adolescents has been controversial and the findings in the literature are inconclusive. Although prior studies have identified several factors that may cause or trigger the proposed relationship, little is known about the culture-related factors as an underlying mechanism that could explain the complexity of this association. This study addressed this gap by examining the associations between the two domains of social media (i.e., time spent on weekdays and weekends) and two mental health outcomes (anxiety and depression) in adolescents via the moderating effect of horizontal-vertical individualism and collectivism. 299 secondary school students (Mage = 15.21 years; 61% girls) from Türkiye (N = 176), Ireland (N = 70), and England (N = 53) completed self-report surveys. Spending more than four hours on weekdays and weekends was positively associated with anxiety and depression whereas spending more than two hours on social media during weekends was positively associated with anxiety in the entire sample. Horizontal and vertical individualism moderated the association between time spent on social media and depression in the entire sample. The study’s strengths and limitations, along with the implications of the findings for future research, are thoroughly discussed.

## Introduction

Adolescence is a vulnerable period for the onset of internalizing symptoms, such as depression and anxiety. The increased popularity of social media and the increased time adolescents spent on social media has led to concerns about whether social media use influences the mental health of adolescents. Research on the influence of social media use on adolescent mental health is extensive and continues to increase. The findings regarding this issue are contradictory and inconclusive in the literature, for instance a recent systematic review examined the findings of 13 studies, 12 of which were cross-sectional, and found that the results of these studies were not entirely consistent for the time spent on social media, and adolescents’ activities on social media, with most authors highlighting that the observed relationship was too complex for straightforward conclusions [1]. The diversity of social media activities, such as active versus passive use, which has been addressed in only a limited number of studies, further complicates this issue. This variation makes it difficult to draw definitive conclusions about the impact of specific types of engagement on mental health outcomes based on such a small body of research. Several studies found that social media have a negative impact on adolescent mental health [2–4]. However, other studies found no association [5–7]. Although there has been a proliferation of studies that examined the complexity of the relationship between the amount of time spent on social media and mental health outcomes in adolescents through several confounding, mediating, and moderating factors (e.g., cyberbullying, insomnia, social support, rumination, age, gender) [8–10], culture-related factors have not been afforded the same attention within the literature. In fact, adolescents’ social media use patterns may be dictated by their cultural values, priorities in life, belief systems and thought patterns. The interaction of culture and social media use may then predict the mental health outcomes in adolescents. Therefore, it is important to examine how social media influence adolescent mental health in different cultures.

However, culture is a very complex and a broad concept. Much of our understanding of culture today is based on the findings of the study published by Dutch psychologist Geert Hofstede in 1980. Between 1967 and 1973, Geert Hofstede conducted a large survey on 116,000 IBM employees from over 50 countries, and he compared them in terms of their national values [11]. As a result of factor analyses, he identified five bi-polar cultural dimensions: individualism/collectivism, power distance, masculinity/femininity, uncertainty avoidance and short term-long term orientation [12]. Among the various cultural dimensions, individualism and collectivism stand out as particularly significant and influential, varying not only between countries but also among individuals [12–15]. This dimension, which encompasses differences in emotion, cognition, motivation, and behaviour, holds a prominent place in multiple disciplines, notably psychology [16]. Our study emphasizes individualism-collectivism due to its unique measurability at the individual level compared to other dimensions, and its profound connections to self-concept, cultural values, life priorities, motivations, and psychological behaviours.

According to Hofstede, Western countries such as the United States, Canada, United Kingdom, Ireland, and Australia are considered individualist [11]. Individuals from individualist cultures are autonomous and independent from their in-groups; they place more value on themselves, their personal wants, needs, and goals, against the needs and benefits of their families, friends, and in-groups [17, 18]. In contrast, Asian, South American, African and Middle Eastern countries including China, Korea, Thailand, Brazil, Peru, Iran and Türkiye are considered collectivist [11]. Individuals from collectivist cultures are interdependent to their in-groups (e.g., family, classmates, colleagues, nation); they value the needs and benefits of their in-groups more than their personal wants, needs, and goals [17, 18]. Their relationships with other people and a sense of belonging to an in-group are very important to them and they tend to make fewer but closer and stronger connected friendships [19].

Although Hofstede’s model is still widely used in a range of disciplines, the model is limited to societal level. Triandis conceptualized the dimension in individual level and classified individualism-collectivism as horizontal and vertical [13]. While vertical dimension refers to the ‘different self’ (hierarchy/inequality), the horizontal dimension refers to the ‘same self’ (equality) [13, 20]. Horizontal individualists tend to be highly self-reliant, independent, willing to be unique, but they are not motivated to attain higher status [21]. In contrast, vertical individualists are autonomous and independent, but they are willing to attain a higher status, and desire to be distinguished themselves from others through displaying their status, power, and prestige favourably and attractively to others [22]. Horizontal collectivists are interdependent on each other and have a desire to build benevolent relationships with others and behave socially appropriately [22]. Vertical collectivists are interdependent, but they emphasize the importance of higher status and power [20, 21]. In contrary to vertical individualists, vertical collectivists are willing to sacrifice their personal goals for the benefit of their in-group [22].

### Individualism and collectivism in the context of social media

Studies have identified some differences between individualists and collectivists regarding social media usage patterns, motivations, and online behaviours (e.g., self-disclosure, self-presentation, and social comparison). For instance, a study that compared social media use among Chinese and American college students found that Americans, characterized as individualists, spend more time on social media compared to Chinese users, who are typically collectivist [19]. In regard to the differences in motives for using social media, Sheldon and colleagues found that Croatians, driven by collectivist values, predominantly use Instagram for social interaction, while Americans, driven by individualist values, use Instagram for self-promotion and documentation (e.g., using hashtags) purposes [23]. Another study found diverse motives for social media usage across countries such as Iran, Malaysia, the UK, and South Africa, which were information seeking, interpersonal utility, convenience, and pass time, respectively [24]. Despite Iran and Malaysia sharing collectivist tendencies and the UK and South Africa exhibiting individualist traits, distinct motives for social media use were evident in each context, possibly influenced by the horizontal and vertical dimensions of individualism and collectivism. Moreover, evidence shows a positive association between collectivist orientations and the use of social media to foster bonding and bridging relationships [25, 26].

In regard to the differences in online behaviours between individualists and collectivists, studies found that American social media users tend to engage more in positive self-presentation but disclose less personal information compared to their collectivist counterparts, such as Korean and Chinese users [27, 28]. These findings align with cultural frameworks suggesting that individualist societies value assertiveness and positive self-image more than collectivist societies, where modesty and group harmony may be prioritized [29–31]. In addition, literature findings highlight differences in social comparison tendencies between individualists and collectivists. For instance, a study by Mao found that Americans favour downward social comparison with acquaintances, whereas Chinese users tend toward upward social comparison with higher achievers [32].

In summary, it has been shown from the existing evidence that individuals’ social media use patterns may be influenced by their cultural orientation, and thus the interaction between culture and social media use may predict mental health outcomes.

### The current study

The overarching aim of the current study was to examine the association between time spent on social media and mental health outcomes (i.e., anxiety and depression) via the moderating effect of vertical and horizontal cultural dimensions of individualism and collectivism. The study’s hypotheses have been grouped together under the two main aims.

#### Social media use and mental health

The first aim was to examine the association between social media use (time spent weekday and weekend) and two mental health outcomes (anxiety and depression) in adolescents. As discussed earlier, previous studies in the literature revealed mixed and inconsistent findings on the relationship between time spent on social media and mental health outcomes among adolescents. As there are ambiguous findings in the literature, we aimed to explore this relationship further. The time spent variable was separated by weekdays and weekends. This was because the target samples were of school age, it was expected that the time they spend on social media during school days and weekends might differ in a way that they would spend more time on social media during weekends.

H1a: There will be an association between time spent on social media (spent hours in the weekday and weekend) and anxiety after having controlled for other variables.
H1b: There will be an association between time spent on social media (spent hours in the weekday and weekend) and depression after having controlled for other variables.

#### Social media use, mental health, and individualism-collectivism

The second aim was to examine the moderating effect of horizontal-vertical individualism and collectivism in the relationship between social media use and mental health outcomes in adolescents. In line with the literature findings on the differences in social media use patterns (e.g., time spent, motives for social media use, self-disclosure, self-presentation, social comparison) between individualists and collectivists, we expect that the interaction of social media use and cultural orientation may predict mental health outcomes in adolescents. Therefore, this study hypothesized that:

H2a: Horizontal-vertical individualism and collectivism will moderate the association between time spent on social media (spent hours in the weekday and weekend) and anxiety after having controlled for other variables.
H2b: Horizontal-vertical individualism and collectivism will moderate the association between time spent on social media (spent hours in the weekday and weekend) and depression after having controlled for other variables.

This study focuses on middle adolescents aged 14 to 16, a group particularly significant due to the distinctive developmental stage they are navigating, characterized by peak physical growth, extensive identity exploration, heightened emotional sensitivity, and increased peer influence [33]. During this period, substantial bodily changes can exacerbate concerns about body image, particularly due to the pervasive influence of social media, which often promotes idealized and unrealistic standards of beauty. Middle adolescents frequently depend on concrete thinking [34], especially in stressful situations, which can lead to impulsive decision-making and an increase in risk-taking behaviours, further exacerbated by the influence of social media that often reinforces immediate gratification and peer validation.

Furthermore, this developmental phase is marked by a pronounced quest for autonomy, often resulting in conflicts with parents, heightened peer pressure, a strong desire for social acceptance, efforts to conform, the emergence of romantic relationships, and notable mood fluctuations [33]. The impact of social media can intensify these dynamics, as adolescents may feel additional pressure to present themselves in certain ways online and compare their lives to the curated images of their peers. As these adolescents grapple with the complexities of physical, social, and psychological changes, they may experience significant stress and find it challenging to regulate their intense emotions. Consequently, the interplay of physical changes, cognitive development, emotional regulation, and social dynamics renders this age group particularly vulnerable to mental health challenges. By exploring this critical age range, the study seeks to offer nuanced insights into the intricate relationship between social media use and mental health. The goal is to inform the development of more effective interventions and support strategies tailored to adolescents during this pivotal stage of their lives.

By investigating this critical age range, the study aims to provide nuanced insights into the association between time spent on social media and mental health outcomes, while examining the moderating effects of vertical and horizontal cultural dimensions of individualism and collectivism. The goal is to inform the development of more effective interventions and support strategies tailored to adolescents during this pivotal stage of their lives.

## Methods

This was a cross-sectional study that examined anonymous data from secondary schools in Türkiye, England and Ireland, and carried out between 1^st^ January 2020 and 1^st^ July 2021.

The research is reported in accordance with the Checklist for Reporting Results of Internet E-surveys (CHERRIES) statement [35].

### Ethical approval

Ethical approval for this study was granted by the Ethics Committee from the Psychiatry, Nursing and Midwifery Research Ethics Subcommittee at King’s College London (Ref: HR-19/20-11454). Due to the Covid-19 pandemic and the consequences of restrictions put in place by the UK governments, some changes in data collection methods have been made and full ethical approval was granted by the ethics committee at King’s College London (RESCM-20/21-11454). These adjustments included modifications to the sample recruitment and data collection procedures in response to the challenges posed by the pandemic. The early closure of schools, teachers’ difficulties in adapting to remote teaching, and their reluctance to take on additional workload significantly hindered access to the target population in the UK. Consequently, we opted to employ convenience and network sampling methods, expanding the research sites to include both England and the Republic of Ireland. Additionally, in light of COVID-19 restrictions, we had to transition from traditional paper-and-pen data collection to an online survey format.

Alongside ethical approval, permission to conduct research in Türkiye was obtained from the General Directorate of Innovation and Educational Technologies. As participants were recruited from schools, written permission from school headteachers to approach potential participants was obtained. The lead author (BK) obtained written informed assent from all participants and a written consent was also obtained from parents/legal guardians of participants aged 14–15 years prior to participation in this study.

### Participants

Participants were recruited from Türkiye, Ireland, and England. According to Hofstede’s cultural dimension scores, at a score of 37 Türkiye is a collectivist country (below the cut-off point of 50) whereas the United Kingdom and Ireland are individualist countries at a score of 89 and 70 respectively [12]. These countries were selected to increase the possibility of recruiting both collectivist and individualist adolescents for this study in compliance with the aim of the study.

All participants were recruited from secondary schools. Schools in Türkiye were randomly selected from an exhaustive list of four types of schools in Diyarbakir, Türkiye. For permission to approach schools in Türkiye, approval was granted by the General Directorate of Innovation and Educational Technologies in Türkiye. All schools in Diyarbakir, Türkiye were stratified by the four types of school (i.e., Anatolian high school that refers to a public school in Türkiye that admit their students according to their test scores in a nationwide exam; vocational and technical Anatolian school; faith school; and private school) and one school was randomly selected from each stratum. Randomly selected schools were visited in person by a member of the research team (the first author) in January 2020 and invited to take part in the study. All visited schools (N = 4) agreed to participate.

Schools in Ireland and England were selected using convenience and network sampling in the Spring of 2021 due to the challenges faced during the Covid-19 pandemic. To identify these schools, we used contacts of one of the authors who had existing relationships with either these schools or the academy trust that is responsible with these schools. Schools in Ireland were contacted via email and phone calls, while schools in England were contacted via E-ACT, which is a multi-academy trust responsible for 28 academies in England. Although numerous schools have been contacted and invited to take part, only three schools from England, one of which was a private and very small school, and two schools from Ireland have participated in the study.

Participants were eligible if they (a) were between 14 and 16 years old, (b) had capacity to give informed consent, and (c) have provided assent/consent along with parental consent if needed. Students were excluded if they (a) did not give assent, (b) did not get consent from their parents, and (c) did not complete more than two core measures (i.e., anxiety, depression, and culture scales).

### Procedure

The following procedure was followed to collect data in Türkiye. With the assistance of the principals and respective teachers, a study pack, which included a Participant Information Sheet describing the study aim and procedure, an assent form, a parental consent form, and a questionnaire booklet, were distributed to each student from two classrooms in each school. Students were asked to take the study packs to their home, read the information sheet, get consent from their parents, complete their own assent form/s, and the questionnaire booklet, and return them to the responsible teacher at school.

Data from England and Ireland was collected through anonymous online survey. The online survey was administered through an Internet-based survey website called ‘Qualtrics’, taking approximately 15–20 minutes to complete. Before fielding the questionnaire, the usability and technical functionality of the electronic questionnaire had been tested with the research team. Once the head teacher at every school agreed to take part in the study, they were provided with two anonymous links; one was for the informed parental consent and the other was for the informed participant assent/consent and the survey. The principals/respective teachers were asked to distribute these links to the parents of 14-15-year-olds and separate link to students aged 16 years via email. If the students aged 16 years were interested in taking part, they were asked to complete the consent form and the online survey. If the parents/legal guardians of the students aged 14–15 years were interested in their child’s participation in the study, they were required to complete the consent form and ask their child to complete the online survey. The online survey consisted of nine pages including an information page, an assent/consent page, an instruction page, and six pages of the questionnaire. The number of questionnaire items per page ranged from eight to sixteen. Those who clicked on the survey link were directed to the information page at the beginning of the survey. Assent/Consent was obtained from all the students when they read the statements and agreed to complete the questionnaires through an online form at the beginning of the survey. It was also made clear that participating was voluntary and completing the survey would mean they were consenting to take part in this study. While completing the survey, respondents were able to review and make changes to their answers using a back button. To prevent any duplicate entries, the ‘closed’ survey was never displayed a second time once the respondent had completed it. Participants did not receive any incentives/reward for participating in this study.

### Measures

#### Individualism and collectivism-16 (INDCOL-16)

Individual level individualism and collectivism Scale [21] was used to measure individuals’ cultural orientations. The Individualism and Collectivism scale (INDCOL-16) is a 16-item scale, and the original scale was divided into four sub-scales each of which involves 4 items. These sub-scales measured ‘Vertical Collectivism’ (VC; example item: “It is my duty to take care of my family, even when 1 have to sacrifice what I want”), ‘Vertical Individualism’ (VI; e.g., “Winning is everything”), ‘Horizontal Collectivism’ (HC; e.g., “I feel good when I cooperate with others.”) and ‘Horizontal Individualism’ (HI; e.g., “I often do "my own thing."). All items were answered on a 9-point scale, response options ranging from 1 = strongly disagree and 9 = strongly agree. The scale was translated into the Turkish language using the back-translation method by Li and Aksoy [36]. We only replaced the word ‘co-workers’ with ‘classmates’ which was more appropriate for school age adolescents. To replicate the factor structure of the INDCOL-16 scale and to test the validity and reliability of the scale, we ran both EFA and CFA using a combined data from Turkish and English-speaking samples. These analyses revealed that the fourth statement of the Vertical Collectivism subscale (labelled as VC4) loaded on the HC factor rather than the expected factor. When VC4 was removed from the analysis, the modified model provided a better fit to data (XXX., under review). Cronbach’s Alpha scores for HC, VC, VI, and HI were 0.823, 0.771, 0.766, 0.748 respectively for English version, 0.695; 0.714; 0.690; and 0.640 for the Turkish version.

#### Anxiety symptoms

The 7-item Generalized Anxiety Disorder Scale (GAD-7) [37] was used to measure the severity of anxiety symptoms. All items were answered on a 4-point scale, response options ranging from 0 (not at all) and 3 (nearly every day). The total scores ranged from 0 to 21; scores of 5, 10 and 15 represent the thresholds for mild, moderate, and severe anxiety respectively and were used to create a four-category ordinal variable. A cut-off point of 10 has been used to differentiate anxiety and non-anxiety [37]. The GAD-7 scale has been validated in Turkish by Konkan et al. and found reliable with a Cronbach’s alpha of 0.852 [38]. In the current study, the Cronbach’s alpha coefficients of reliability were calculated as 0.897 for the English version and 0.864 for the Turkish version.

#### Depressive symptoms

The Patient Health Questionnaire 9-item depression scale (PHQ-9) [39] was used to measure the severity of depressive symptoms based on the Diagnostic and Statistical Manual of Mental Disorders, 4th edition (DSM-IV) diagnostic criteria. All items were answered on a 4-point scale, response options ranging from 0 (not at all) and 3 (nearly every day). The total scores ranged from 0 to 27; scores of 5, 10, 15, and 20 represent the thresholds for mild, moderate, moderately severe, and severe depression respectively and were used to create a five-category ordinal variable [39]. A cut-off point of 10 has been used to differentiate depression and non-depression [40]. The scale has been validated in Turkish by Sari et al. and found reliable with a Cronbach’s alpha of 0.842 [41]. The PHQ-9 demonstrated an excellent internal reliability in the current study with a Cronbach’s alpha of 0.907 for the English version and 0.841 for the Turkish version.

#### Social media use

*(1) Time spent on social media*. Time spent on social media was separated into two time periods, weekday and weekend, because screen time may differ between weekdays (school days) and weekends (non-school days) among adolescents. Time spent on social media was measured with two items, “In the past weekdays, on average, approximately, how much time per day have you spent on social media?” and “In the past weekend, on average, approximately, how much time per day have you spent on social media?”. Four options were given for each item: hardly ever, 1–2 hours a day, 2–4 hours a day, and more than 4 hours a day.

*(2) General questions about social media*. Participants were also asked questions about their access to internet, whether they own a smartphone, the length of social media membership, the type of device they use to access social media, which social media platforms they use the most, the average number of followers, the types of people they follow (e.g., relatives, friends, strangers) and the types of people their followers are. They were also asked to what extent social media has integrated into their daily life.

#### Demographic information

Demographic information was collected via a short questionnaire that consists of twelve questions, three of which were open-ended questions and were used to obtain a participants’ age, country of origin, and the number of siblings. Age, gender, country of origin, the type of school they were registered in, the number of siblings in the family, parents’ educational level and occupation were included in the model as control variables.

### Sample size

The plan in the original study protocol was to recruit about 200 adolescents from each country [42]. To test for an R^2^ change of 5%, with power of 80% at the 5% level of significance, after the addition of a single independent variable, would require a sample of 136. This number was increased to 200 to account for missing data.

### Statistical analysis

Survey data were described statistically using frequencies and percentages. Prior to undertaking the hierarchical regression analysis consideration was given to missing data. The proportion of missing data for the model variables ranged between 0 to 12% therefore a complete case analysis, that only uses responders with no missing data, would have led to a reduced sample [43]. To minimise any potential biases caused by missing data, we therefore conducted a Multiple Imputation using Chained Equations (MICE). The imputation model included all variables used in the hierarchical regression [44]. Imputed datasets were created using a method appropriate for data consisting of multiple psychometric scales, in our case INDCOL, GAD7 and PHQ9 [45]. A total of 40 imputed datasets were generated which was sufficient to meet the largest fraction of missing information (FMI) criterion (0.37 x 100 = 37) for all the fitted models [46]. Following the imputation collectivism was classified into four groups using VC and HC mean scores (high VC and high HC; high VC and low HC; low VC and high HC; low VC and low HC) and individualism classified into four groups using VI and HI (high VI and high HI; high VI and low HI; low VI and high HI; low VI and low HI). For each sub-scale mean scores of five or less were allocated to low and greater than five to high.

Hierarchical regression analysis was then used to investigate the direct effects of time spent on social media (weekdays and weekends) on the dependent variables (i.e., anxiety and depression) and the interaction effect between time spent on social media and cultural dimensions. To eliminate the influences of the other variables in these relationships, variables including age, gender, country of residence, type of school, number of siblings, mother’s and father’s educational attainment, occupations, and the number of followers were included in each model as control variables. For example, older age, girls, wealth-poverty, low socioeconomic status (which may be linked to the parent’s educational level and their occupations) can be predictors of poor mental health outcomes [47–50].

To assess the effects of the time spent on social media and moderating variables on GAD7 and PHQ9, variables were added in blocks:

1. control variables and time spent on social media were included in the first step;
2. a single moderating variable (VC, VI, HC or HI) was added next;
3. the interaction effect of time spent on social media and the single moderating variable was added in the final step.

Blocks 2 and 3 were therefore repeated four times for VC, VI, HC and HI. VC, VI, HC and HI were not fitted simultaneously due to concerns relating to collinearity, overfitting and to provide clearer insights of how each cultural dimension influences the relationship between social media use and mental health outcomes. The analyses were also performed separately for time spent on weekdays and weekends to avoid collinearity between two potentially highly correlated variables. There were no statistically significant interactions involving anxiety, so our reporting is confined to depression only.

A similar approach was followed for the collectivism and individualism classifications with collectivism (or individualism) added at block 2 and the interaction effect of time spent on social media and collectivism (or individualism) added at block 3. There were no statistically significant relationships between these classification variables and anxiety and depression. Therefore, they have not been reported in the results and the analysis focuses specifically on the individual INDCOL sub-scales (VC, VI, HC or HI).

For the time spent on social media, responses on “hardly ever” and “1–2 hours” (weekdays and weekends) were grouped together for the England-Ireland sample because there were only 8 and 9 respondents who responded as "hardly ever" for time spent on social media weekdays and weekends respectively.

Analyses were performed on the entire sample (that included a variable for country of residence in the model) and then separately for Türkiye (collectivist), and England-Ireland combined (individualistic). The intention had been to analyse England and Ireland separately, but their respective sample sizes were considered too small. However, they were combined on the basis that they shared the same language and had similar cultures.

The overall effect of time spent on social media and interactions with the INDCOL sub-scales were tested using a F Statistic that was calculated from the multiply imputed data. The denominator degrees of freedom can be non-integer and are shown to one decimal point. Each ordinal category of time spent on social media was statistically compared against the ‘Hardly ever’ category for the overall and Turkish samples and the against ‘Less than two hours a day’ category for the English Irish sample. The β estimate, 95% confidence interval and p-value have been presented for each comparison. Where statistically significant interactions were present the model parameter estimates shown in Table 6 have been used to determine which individuals are at most and least risk of depression (all interactions with anxiety were not statistically significant).

Consideration was given to calculating pooled standardised beta estimates derived from the analysis of the imputed data. van Ginkel [51] proposed two combination rules for pooling because none currently exist. These produce pooled standardized coefficients with small bias however, as far we are aware, these approaches have not yet been implemented into Stata or other mainstream statistical software. It is also noted that neither approach satisfies the four conditions for standardized regression coefficients, one approach satisfies three of the conditions and the other only two. We have therefore presented unstandardized beta coefficients.

The descriptive analysis was produced using the Statistical Package for the Social Sciences (SPSS) version 26.0 (Armonk, NY, USA). The multiple imputation and hierarchical regression analyses were performed using Stata V18.0 (StataCorp, College Station, TX, USA).

## Results

### Descriptive analysis

**Fig 1:**
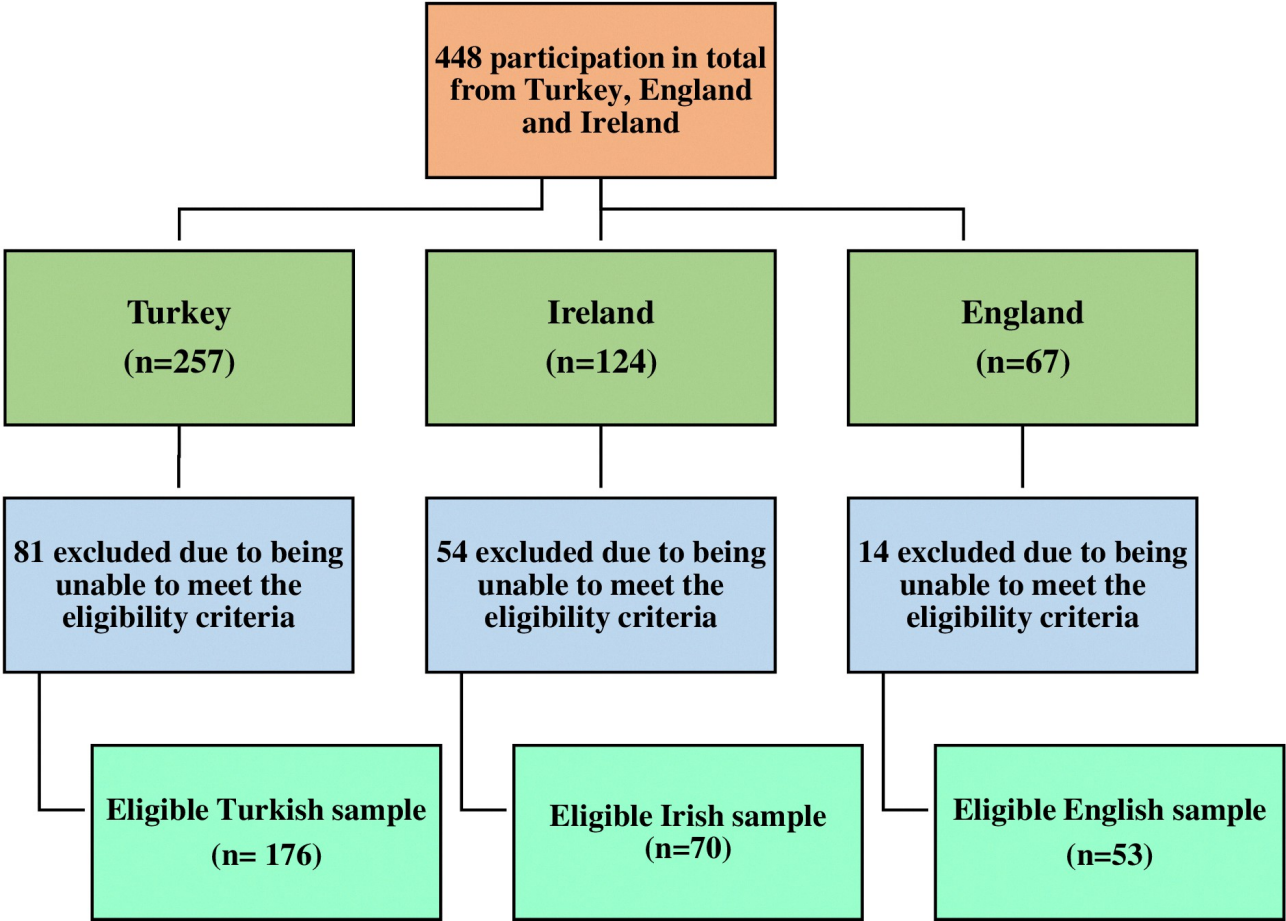
Breakdown of the eligible survey respondents for each county.

A total of 448 secondary school students from Türkiye, England and Ireland were invited to participate. Forty-seven of these students did not give assent/consent to participate and/or did not get consent from their parents. Data of seven students could not be used because they did not meet the age criteria (14–16 years). Ninety-five students did not complete the survey or did not complete more than two core measures. The final sample reduced to 299 (M_age_ = 15.21 years; SD = 0.74; 61% girls), consisting of 176 participants from Türkiye, 70 from Ireland and 53 from England. They were from four schools in Türkiye (Diyarbakir), two schools in Ireland (Dublin and Galway), and three schools in England (Manchester, Sheffield, and Verwood). The flowchart below (Fig 1) provides further detail on the total number of survey respondents and the number of included and excluded respondents for each country.

Table 1 below demonstrates the descriptive characteristics of the sample.

**Table 1:** Descriptive characteristics of the sample.

Variable	Country of residence			
	**Türkiye**	**Ireland**	**England**	**Total**
**N**	176	70	53	299
**Age**				
14	32 (18.2%)	18 (25.7%)	5 (9.4%)	55 (18.4%)
15	88 (50.0%)	24 (34.3%)	11 (20.8%)	123 (41.1%)
16	53 (30.1%)	28 (40.0%)	37 (69.8%)	118 (39.5%)
**Gender**				
Male	59 (33.5%)	38 (54.3%)	21 (39.6%)	118 (39.5%)
Female	117 (66.5%)	32 (45.7%)	32 (60.4%)	181 (60.5%)
**Mother’s educational attainment**				
No education	58 (33.0%)	-	10 (18.9%)	68 (22.7%)
Primary school	83 (47.2%)	-	2 (3.8%)	85 (28.4%)
Secondary/high school	28 (15.9%)	17 (24.3%)	17 (32.1%)	62 (20.7%)
College/University	5 (2.8%)	52 (74.3%)	23 (43.4%)	80 (26.8%)
**Mother’s occupation**				
Unemployed	164 (93.2%)	6 (8.6%)	29 (54.7%)	199 (66.6%)
Employed/Self-employed	8 (4.5%)	61 (87.1%)	23 (43.4%)	92 (30.8%)
Retired	1 (0.6%)	2 (2.9%)	-	3 (1.0%)
Other (unknown, no mother)	1 (0.6%)	1 (1.4%)	1 (1.9%)	3 (1.0%)
**Father’s educational attainment**				
No education	6 (3.4%)	1 (1.4%)	8 (15.1%)	15 (5%)
Primary school	86 (48.9%)	5 (7.1%)	-	91 (30.4%)
Secondary/high school	50 (28.4%)	26 (37.1%)	23 (43.4%)	99 (33.1%)
College/University	32 (18.2%)	38 (54.3%)	21 (39.6%)	91 (30.4%)
**Father’s occupation**				
Unemployed	18 (10.2%)	1 (1.4%)	9 (17.0%)	28 (9.4%)
Employed/Self-employed	140 (79.5%)	66 (94.3%)	37 (69.8%)	243 (81.3%)
Retired	10 (5.7%)	1 (1.4%)	4 (7.5%)	15 (5.0%)
Other (unknown/no father)	2 (1.1%)	2 (2.9%)	3 (5.7%)	7 (2.3%)
**Access to the internet**				
No access	7 (4.0%)	-	-	7 (2.3%)
Via home broadband	94 (53.4%)	55 (78.6%)	49 (92.5%)	198 (66.2%)
Via mobile data	60 (34.1%)	15 (21.4%)	4 (7.5%)	79 (26.4%)
Via public Wifi	13 (7.4%)	-	-	13 (4.3%)
**Smartphone ownership**				
Yes	86 (48.9%)	68 (97.1%)	49 (92.5%)	203 (67.9%)
No	90 (51.1%)	2 (2.9%)	4 (7.5%)	96 (32.1%)
**Used device to access social media**				
Smartphone	143 (81.2%)	67 (95.7%)	48 (90.6%)	258 (86.3%)
Tablet	14 (8.0%)	2 (2.9%)	2 (3.8%)	18 (6.0%)
Personal computer	5 (2.8%)	1 (1.4%)	3 (5.7%)	9 (3.0%)
Public computer	7 (4.0%)	-	-	7 (2.3%)
**Time spent on social media (weekday)**				
Hardly ever	51 (29.0%)	6 (8.6%)	2 (3.8%)	59 (19.7%)
1–2 hours a day	81 (46.0%)	19 (27.1%)	10 (18.9%)	110 (36.8%)
2–4 hours a day	31 (17.6%)	27 (38.6%)	17 (32.1%)	75 (25.1%)
More than 4 hours a day	13 (7.4%)	18 (25.7%)	23 (43.4%)	54 (18.1%)
**Time spent on social media (weekend)**				
Hardly ever	43 (24.4%)	7 (10.0%)	2 (3.8%)	52 (17.4%)
1–2 hours a day	51 (29.0%)	13 (18.6%)	10 (18.9%)	74 (24.7%)
2–4 hours a day	47 (26.7%)	28 (40.0%)	17 (32.1%)	92 (30.8%)
More than 4 hours a day	35 (19.9%)	22 (31.4%)	23 (43.4%)	80 (26.8%)

Seven participants (n = 7) from Türkiye reported having no internet access; however, they were not excluded from the analysis, as limited or no internet access does not necessarily preclude the possibility of engaging with social media. Additionally, the study’s inclusion criteria did not mandate that participants have internet access or be active social media users, which allowed the inclusion of individuals who rarely use social media alongside those who are frequent users. This approach ensured a comprehensive representation of the population and enhanced the analysis by incorporating a broader range of experiences related to adolescent mental health, regardless of the participants’ level of social media engagement.

YouTube (68%), Instagram (65%), and Snapchat (45%) were the most popular social media platforms among all participants, which were followed by Pinterest (11.7%), Twitter (11.4%), Facebook (8.4%), TikTok (7.4%), Tumblr (0.7%), and Myspace (0.7%). Snapchat was specifically more popular among Irish (71%) and English (70%) participants as opposed to Turkish participants (27%), and YouTube was more popular among Turkish (74%) and English (72%) participants as opposed to Irish participants (47%).

Table 2 shows the severity score distribution for the anxiety and depression for the present study. According to these self-report measures, 42% of the total had moderate to severe anxiety, and 44% had moderate to severe depression. Moderate to severe anxiety and depression were more common among Turkish participants (47% and 53% respectively) compared to Irish (36%, 31%) and English participants (34%, 34%).

**Table 2:** Score distribution for anxiety and depression scale by severity.

	Türkiye (N = 176)	Ireland (N = 70)	England (N = 53)	Total (N = 299)
**Anxiety**				
**Minimal**	41 (23.3%)	27 (38.5%)	13 (24.5%)	81 (27.2%)
**Mild**	36 (20.4%)	12 (17.1%)	18 (33.9%)	66 (22.1%)
**Moderate**	42 (23.8%)	14 (19.9%)	7 (13.2%)	63 (21.1%)
**Severe**	40 (22.7%)	11 (15.7%)	11 (20.9%)	62 (20.6%)
**Depression**				
**No depression**	27 (15.3%)	24 (34.4%)	13 (24.6%)	64 (21.5%)
**Mild**	36 (20.4%)	13 (18.5%)	17 (32.1%)	66 (22.1%)
**Moderate**	44 (24.9%)	11 (15.8%)	5 (9.5%)	60 (20.1%
**Moderately severe**	33 (18.7%)	6 (8.6%)	6 (11.4%)	45 (15.1%)
**Severe**	16 (9.1%)	5 (7.1%)	7 (13.3%)	28 (9.3%)

### Main findings

#### Association between time spent on social media and mental health outcomes (H1a/b)

As shown in Table 3 below, anxiety and depression levels did not vary significantly between the three countries for weekdays and weekends.

**Table 3:** Overall sample, hierarchical regression models (blocks 1 & 2).

	Weekdays						Weekends					
	Anxiety			Depression			Anxiety			Depression		
	B	p	(95%CI)	B	p	(95%CI)	B	p	(95%CI)	B	p	(95%CI)
**BLOCK 1**	** **	** **										
**Country of residence**	** **	0.26			0.31			0.24			0.38	
Time spent on social media	** **	0.034			0.001			0.004			0.014	
1–2 hours a day	0.73	0.45	(-1.17 to 2.62)	0.96	0.37	(-1.16 to 3.08)	0.89	0.41	(-1.20 to 2.98)	-0.36	0.77	(-2.76 to 2.04)
2–4 hours a day	0.66	0.56	(-1.59 to 2.91)	0.70	0.58	(-1.78 to 3.19)	2.37	0.026	(0.28 to 4.46)	1.04	0.39	(-1.32 to 3.40)
>4 hours a day	3.41	0.008	(0.89 to 5.93)	5.13	<0.001	(2.30 to 7.96)	3.76	0.001	(1.53 to 6.00)	3.20	0.013	(0.69 to 5.71)
**BLOCK2**	** **	** **										
**Vertical Collectivisim**	** **	** **										
**Time spent on social media**	** **	0.030			0.002			0.004			0.024	
1–2 hours a day	0.73	0.45	(-1.17 to 2.62)	0.96	0.37	(-1.16 to 3.08)	0.85	0.42	(-1.24 to 2.95)	-0.28	0.82	(-2.69 to 2.12)
2–4 hours a day	0.69	0.55	(-1.56 to 2.95)	0.63	0.62	(-1.85 to 3.11)	2.38	0.026	(0.29 to 4.47)	1.02	0.39	(-1.33 to 3.38)
>4 hours a day	3.50	0.007	(0.96 to 6.04)	4.92	0.001	(2.09 to 7.76)	3.81	0.001	(1.57 to 6.05)	3.08	0.017	(0.56 to 5.60)
**VCMean**	0.10	0.60	(-0.28 to 0.48)	-0.23	0.29	(-0.66 to 0.20)	0.10	0.59	(-0.27 to 0.48)	-0.25	0.26	(-0.69 to 0.18)
**Horizontal Collectivisim**												
**Time spent on social media**		0.022			0.001			0.004			0.017	
1–2 hours a day	0.61	0.53	(-1.30 to 2.52)	0.98	0.37	(-1.16 to 3.12)	0.81	0.45	(-1.29 to 2.92)	-0.28	0.82	(-2.70 to 2.14)
2–4 hours a day	0.51	0.66	(-1.75 to 2.78)	0.73	0.57	(-1.79 to 3.24)	2.30	0.032	(0.20 to 4.39)	1.12	0.35	(-1.25 to 3.49)
>4 hours a day	3.54	0.006	(1.02 to 6.06)	5.11	<0.001	(2.28 to 7.94)	3.77	0.001	(1.53 to 6.00)	3.20	0.013	(0.69 to 5.71)
**HCMean**	0.20	0.30	(-0.18 to 0.59)	’-0.03	0.89	(-0.46 to 0.40)	0.12	0.54	(-0.26 to 0.49)	-0.13	0.55	(-0.55 to 0.30)
**Vertical Individualism**												
**Time spent on social media**		0.034			0.001			0.008			0.019	
1–2 hours a day	0.56	0.56	(-1.33 to 2.44)	0.76	0.48	(-1.35 to 2.87)	0.71	0.50	(-1.38 to 2.79)	-0.60	0.62	(-3.00 to 1.79)
2–4 hours a day	0.17	0.88	(-2.11 to 2.45)	0.11	0.93	(-2.41 to 2.62)	2.06	0.054	(-0.04 to 4.16)	0.62	0.61	(-1.76 to 2.99)
>4 hours a day	3.12	0.015	(0.61 to 5.64)	4.78	0.001	(1.96 to 7.61)	3.53	0.002	(1.30 to 5.77)	2.89	0.025	(0.37 to 5.40)
**VIMean**	0.40	0.032	(0.04 to 0.77)	0.49	0.020	(0.08 to 0.90)	0.33	0.069	(-0.03 to 0.70)	0.45	0.032	(0.04 to 0.87)
**Horizontal Individualism**												
**Time spent on social media**		0.046			0.001			0.009			0.025	
1–2 hours a day	0.50	0.60	(-1.36 to 2.37)	0.70	0.51	(-1.38 to 2.79)	0.76	0.47	(-1.30 to 2.82)	-0.52	0.67	(-2.87 to 1.84)
2–4 hours a day	0.21	0.85	(-2.02 to 2.44)	0.18	0.89	(-2.27 to 2.63)	2.18	0.038	(0.12 to 4.24)	0.81	0.49	(-1.51 to 3.13)
>4 hours a day	2.99	0.018	(0.51 to 5.47)	4.64	0.001	(1.84 to 7.44)	3.46	0.002	(1.26 to 5.66)	2.83	0.025	(0.36 to 5.31)
**HIMean**	0.64	0.001	(0.25 to 1.03)	0.75	0.001	(0.31 to 1.19)	0.61	0.002	(0.22 to 0.99)	0.74	0.001	(0.30 to 1.18)

Note: Base level for time spent on social media is ‘Hardly ever’, B = 0

Time spent on social media, once it reaches four or more hours a day, significantly increases anxiety and depression compared to adolescents who hardly ever use social media. The effect on depression was more noticeable for weekday use (β = 5.13, 95%CI 2.30 to 7.96, p<0.001) compared to weekends (β = 3.20, 95%CI 0.69 to 5.71, p = 0.013). Neither vertical or horizontal collectivism were associated with anxiety or depression. Vertical and horizontal individualism were both significantly associated with anxiety and depression, the latter was stronger (0.61 to 0.75 increase in anxiety or depression for every one-point increase in mean horizontal individualism) than the former (0.33 to 0.49 increase). Vertical individualism was not significantly associated with anxiety at weekends (β = 0.33, 95%CI -0.03 to 0.70, p = 0.069) although the association was positive.

The association between anxiety and time spent on social media for the Turkish sample (Table 4) was not statistically significant for weekdays (F(3, 151.2) = 0.89, p = 0.45) however it was statistically significant at weekends (F(3, 149.8) = 3.48, p = 0.018).

**Table 4:** Turkish sample, hierarchical regression models (blocks 1 & 2).

	Weekdays						Weekends					
	Anxiety			Depression			Anxiety			Depression		
	B	p	(95%CI)	B	p	(95%CI)	B	p	(95%CI)	B	p	(95%CI)
**BLOCK 1**												
**Time spent on social media**		0.45			0.051			0.018			0.034	
1–2 hours a day	0.14	0.90	(-2.02 to 2.31)	1.21	0.30	(-1.11 to 3.54)	0.43	0.73	(-1.99 to 2.84)	-0.55	0.68	(-3.20 to 2.09)
2–4 hours a day	1.31	0.38	(-1.62 to 4.23)	2.24	0.16	(-0.87 to 5.36)	2.93	0.022	(0.42 to 5.43)	2.50	0.073	(-0.23 to 5.23)
>4 hours a day	2.67	0.16	(-1.09 to 6.42)	5.56	0.006	(1.62 to 9.50)	3.54	0.015	(0.71 to 6.38)	2.79	0.072	(-0.25 to 5.84)
**BLOCK2**												
***Vertical Collectivisim***												
**Time spent on social media**		0.50			0.068			0.023			0.050	
1–2 hours a day	0.11	0.92	(-2.05 to 2.27)	1.17	0.32	(-1.14 to 3.48)	0.50	0.68	(-1.91 to 2.91)	-0.45	0.74	(-3.08 to 2.19)
2–4 hours a day	1.27	0.39	(-1.65 to 4.19)	2.19	0.17	(-0.91 to 5.29)	2.92	0.023	(0.41 to 5.42)	2.48	0.073	(-0.24 to 5.20)
>4 hours a day	2.47	0.20	(-1.29 to 6.23)	5.29	0.009	(1.35 to 9.22)	3.43	0.018	(0.59 to 6.26)	2.62	0.090	(-0.42 to 5.65)
**VCMean**	-0.32	0.21	(-0.83 to 0.18)	-0.44	0.11	(-0.97 to 0.09)	-0.28	0.26	(-0.77 to 0.21)	-0.42	0.12	(-0.96 to 0.11)
***Horizontal Collectivisim***												
**Time spent on social media**		0.47			0.056			0.017			0.035	
1–2 hours a day	0.17	0.88	(-2.01 to 2.34)	1.23	0.30	(-1.10 to 3.56)	0.47	0.70	(-1.95 to 2.88)	-0.52	0.70	(-3.17 to 2.14)
2–4 hours a day	1.36	0.36	(-1.58 to 4.30)	2.28	0.15	(-0.85 to 5.41)	2.96	0.021	(0.45 to 5.47)	2.53	0.069	(-0.20 to 5.26)
>4 hours a day	2.57	0.18	(-1.23 to 6.37)	5.50	0.007	(1.51 to 9.48)	3.56	0.014	(0.72 to 6.40)	2.81	0.071	(-0.24 to 5.86)
**HCMean**	-0.10	0.69	(-0.58 to 0.38)	-0.06	0.80	(-0.57 to 0.44)	-0.14	0.56	(-0.59 to 0.32)	-0.13	0.61	(-0.62 to 0.37)
***Vertical Individualism***												
***Time spent on social media***		0.76			0.17			0.047			0.083	
1–2 hours a day	-0.01	1.00	(-2.15 to 2.14)	1.04	0.37	(-1.25 to 3.32)	0.22	0.86	(-2.17 to 2.60)	-0.85	0.52	(-3.46 to 1.76)
2–4 hours a day	0.67	0.65	(-2.28 to 3.63)	1.49	0.35	(-1.62 to 4.60)	2.53	0.048	(0.02 to 5.04)	1.95	0.16	(-0.77 to 4.67)
>4 hours a day	1.84	0.34	(-1.94 to 5.62)	4.58	0.024	(0.62 to 8.54)	3.04	0.036	(0.20 to 5.88)	2.10	0.17	(-0.93 to 5.13)
**VIMean**	0.52	0.033	(0.04 to 0.99)	0.61	0.015	(0.12 to 1.10)	0.46	0.050	(0.00 to 0.91)	0.63	0.010	(0.15 to 1.12)
***Horizontal Individualism***												
**Time spent on Social media**		0.63			0.10			0.027			0.049	
1–2 hours a day	0.01	0.99	(-2.15 to 2.16)	1.08	0.36	(-1.23 to 3.38)	0.39	0.75	(-2.00 to 2.78)	-0.60	0.65	(-3.22 to 2.02)
2–4 hours a day	0.88	0.55	(-2.06 to 3.83)	1.81	0.25	(-1.31 to 4.92)	2.82	0.026	(0.34 to 5.31)	2.38	0.084	(-0.33 to 5.09)
>4 hours a day	2.17	0.26	(-1.60 to 5.94)	5.05	0.013	(1.10 to 9.00)	3.28	0.024	(0.45 to 6.11)	2.49	0.11	(-0.54 to 5.52)
**HIMean**	0.49	0.062	(-0.03 to 1.00)	0.50	0.070	(-0.04 to 1.04)	0.47	0.062	(-0.02 to 0.97)	0.54	0.045	(0.01 to 1.08)

Note: Base level for time spent on social media is ‘Hardly ever’, B = 0

Total GAD7 (anxiety) was 3.54 units higher (95%CI 0.71 to 6.38, p = 0.015) amongst those using social media more than four hours at weekends compared to adolescents who used social media hardly ever. The overall association of time spent on social media and depression fell short of statistical significance for weekdays (F[3, 150.9] = 2.65, p = 0.051) but was significant at weekends (F(3, 149.6) = 2.96, p = 0.034). Total PHQ9 (depression) was 5.56 units higher (95%CI 1.62 to 9.50, p = 0.006) for those using social media for more than four hours compared to those using social media hardly ever on weekdays. The difference between these two usage groups was noticeably smaller at weekends (β = 2.79 95%CI -0.25 to 5.84, p = 0.072).

Anxiety and depression levels did not differ between England and Ireland on either weekdays or at the weekend (Table 5).

**Table 5:** English Irish sample, hierarchical regression models (blocks 1 & 2).

	Weekdays						Weekends					
	Anxiety			Depression			Anxiety			Depression		
	B	p	(95%CI)	B	p	(95%CI)	B	p	(95%CI)	B	p	(95%CI)
**BLOCK 1**												
**Country of residence**		0.27			0.71			0.22			0.68	
**Time spent on social media**		0.13			0.022			0.089			0.034	
2–4 hours a day	-0.01	0.99	(-2.93 to 2.90)	-1.11	0.53	(-4.63 to 2.42)	1.57	0.31	(-1.46 to 4.60)	-0.20	0.91	(-3.85 to 3.44)
>4 hours a day	2.74	0.098	(-0.52 to 6.00)	3.76	0.065	(-0.24 to 7.75)	3.61	0.032	(0.32 to 6.91)	4.00	0.050	(0.01 to 8.00)
**BLOCK2**												
***Vertical Collectivisim***												
**Time spent on social media**		0.031			0.019			0.019			0.030	
2–4 hours a day	0.48	0.74	(-2.39 to 3.35)	-1.00	0.58	(-4.55 to 2.56)	1.93	0.20	(-1.03 to 4.90)	-0.12	0.95	(-3.79 to 3.54)
>4 hours a day	3.87	0.021	(0.61 to 7.14)	4.02	0.053	(-0.06 to 8.10)	4.67	0.006	(1.38 to 7.97)	4.25	0.041	(0.17 to 8.34)
**VCMean**	0.69	0.029	(0.07 to 1.30)	0.16	0.69	(-0.61 to 0.92)	0.70	0.025	(0.09 to 1.31)	0.16	0.67	(-0.60 to 0.93)
***Horizontal Collectivisim***												
**Time spent on social media**		0.020			0.029			0.016			0.049	
2–4 hours a day	-0.09	0.95	(-2.95 to 2.76)	-1.12	0.53	(-4.66 to 2.42)	1.53	0.31	(-1.44 to 4.49)	-0.21	0.91	(-3.87 to 3.45)
>4 hours a day	4.00	0.019	(0.68 to 7.33)	3.94	0.062	(-0.21 to 8.08)	4.83	0.006	(1.45 to 8.21)	4.12	0.054	(-0.07 to 8.30)
**HCMean**	0.75	0.042	(0.03 to 1.48)	0.10	0.82	(-0.78 to 0.99)	0.73	0.049	(0.00 to 1.46)	0.07	0.88	(-0.83 to 0.96)
***Vertical Individualism***												
***Time spent on social media***		0.12			0.018			0.089			0.030	
2–4 hours a day	-0.09	0.95	(-3.05 to 2.87)	-1.31	0.47	(-4.89 to 2.26)	1.53	0.33	(-1.55 to 4.61)	-0.41	0.83	(-4.13 to 3.30)
>4 hours a day	2.76	0.097	(-0.51 to 6.02)	3.80	0.062	(-0.19 to 7.79)	3.61	0.033	(0.31 to 6.92)	4.00	0.050	(0.00 to 8.00)
**VIMean**	0.12	0.73	(-0.56 to 0.80)	0.31	0.45	(-0.50 to 1.13)	0.05	0.88	(-0.63 to 0.73)	0.27	0.52	(-0.55 to 1.09)
***Horizontal Individualism***												
***Time spent on social media***		0.091			0.012			0.074			0.023	
2–4 hours a day	-0.10	0.94	(-2.92 to 2.72)	-1.22	0.47	(-4.60 to 2.15)	1.47	0.32	(-1.47 to 4.40)	-0.34	0.85	(-3.85 to 3.16)
>4 hours a day	2.78	0.081	(-0.35 to 5.90)	3.80	0.050	(0.00 to 7.61)	3.59	0.027	(0.41 to 6.76)	3.96	0.042	(0.14 to 7.79)
**HIMean**	0.88	0.010	(0.21 to 1.55)	1.18	0.004	(0.39 to 1.96)	0.87	0.011	(0.20 to 1.53)	1.16	0.005	(0.37 to 1.95)

Note: Base level for time spent on social media is ‘< 2 hours’, B = 0

Time spent on social media was not associated with anxiety but was associated with depression on weekdays (F(2, 94.4) = 3.97, p = 0.022) and weekends (F(2, 95.7) = 3.51, p = 0.034). Depression, measured using total PHQ9, was four units higher for adolescents who spent four hours or more compared to less than two hours on social media at weekends (β = 4.00 95%CI 0.01 to 8.00, p = 0.050). The same comparison was not statistically significant for weekdays (3.76 95%CI -0.24 to 7.75, p = 0.065) albeit of a similar order of magnitude.

Vertical and horizontal collectivism was positively associated with anxiety but not depression for weekdays (β = 0.69 95%CI 0.07 to 1.30, p = 0.029 and β = 0.75 95%CI 0.03 to 1.48, p = 0.042 respectively) and weekends (β = 0.70 95%CI 0.09 to 1.31, p = 0.025 and β = 0.73 95%CI 0.00 to 1.46, p = 0.049 respectively). Vertical individualism was not associated with either anxiety or depression whilst horizontal individualism was positively associated with both anxiety and depression on weekdays (β = 0.88 95%CI 0.21 to 1.55, p = 0.010 and β = 1.18 95%CI 0.39 to 1.96 respectively), p = 0.004) and at weekends (β = 0.87 95%CI 0.20 to 1.53, p = 0.011 and β = 1.16 95%CI 0.37 to 1.95, p = 0.005 respectively).

There were no statistically significant interactions for anxiety between the four INDCOL dimensions and time spent on social media. However, there were statistically significant interactions for depression between vertical individualism and time spent on social media for the overall sample (F(3, 259.4) = 3.13, p = 0.026), the Turkish (F(3, 139.4) = 2.67, p = 0.050) and the English Irish samples (F(2, 92.7) = 5.82, p = 0.004) on weekdays, and the English Irish sample at weekends (F(2, 92.7) = 6.44, p = 0.002) (Table 6).

**Table 6:** Moderating effect of vertical and horizontal individualism on time spent on social media on depression (Block 3).

	Overall sample			Türkiye				England-Ireland			England-Ireland		
	Weekdays			Weekdays				Weekdays			Weekends		
	B	p	(95%CI)	B	p	(95%CI)		B	p	(95%CI)	B	p	(95%CI)
***Vertical Individualism***							***Vertical Individualism***						
**Time spent on social media**							**Time spent on social media**						
1–2 hours a day	7.48	0.009	(1.88 to 13.09)	9.66	0.003	(3.29 to 16.03)							
2–4 hours a day	1.20	0.73	(-5.62 to 8.01)	-0.03	1.00	(-16.30 to 16.25)	2–4 hours a day	-10.51	0.023	(-19.52 to -1.49)	-11.59	0.015	(-20.82 to -2.35)
More than 4 hours a day	4.59	0.19	(-2.25 to 11.43)	4.23	0.63	(-12.92 to 21.38)	More than 4 hours a day	-10.45	0.026	(-19.61 to -1.30)	-10.65	0.021	(-19.67 to -1.63)
**VIMean**	1.00	0.010	(0.24 to 1.75)	1.43	<0.001	(0.64 to 2.22)	**VIMean**	-1.75	0.019	(-3.21 to -0.30)	-2.06	0.007	(-3.55 to -0.58)
**Time spent on social media x VIMean**		0.026			0.050		**Time spent on social media x VIMean**		0.004			0.002	
1–2 hours a day	-1.20	0.012	(-2.14 to -0.26)	-1.46	0.005	(-2.46 to -0.45)	2–4 hours a day	2.06	0.025	(0.27 to 3.85)	2.52	0.008	(0.68 to 4.35)
2–4 hours a day	-0.22	0.69	(-1.31 to 0.87)	0.07	0.95	(-2.25 to 2.39)	More than 4 hours a day	3.26	0.001	(1.37 to 5.15)	3.41	0.001	(1.51 to 5.32)
More than 4 hours a day	0.05	0.93	(-1.08 to 1.18)	-0.10	0.94	(-2.47 to 2.27)							
***Horizontal Individualism***													
**Time spent on social media**													
1–2 hours a day	8.64	0.018	(1.50 to 15.77)	11.73	0.003	(3.99 to 19.48)							
2–4 hours a day	2.83	0.55	(-6.44 to 12.10)	9.83	0.121	(-2.62 to 22.27)							
More than 4 hours a day	3.22	0.46	(-5.27 to 11.71)	16.58	0.084	(-2.25 to 35.40)							
**HIMean**	1.28	0.005	(0.40 to 2.16)	1.63	0.001	(0.69 to 2.58)							
**Time spent on social media x HIMean**		0.050			0.051								
1–2 hours a day	-1.27	0.026	(-2.38 to -0.15)	-1.72	0.005	(-2.91 to -0.53)							
2–4 hours a day	-0.46	0.52	(-1.85 to 0.94)	-1.33	0.16	(-3.19 to 0.54)							
More than 4 hours a day	0.14	0.83	(-1.12 to 1.40)	-1.80	0.18	(-4.45 to 0.85)							

Note: Base level for time spent on social media for the overall sample and Türkiye is ‘Hardly ever’, B = 0 and for England-Ireland is ‘< 2 hours’, B = 0

Horizontal individualism and time spent on social media interacted significantly for the overall sample (F(3, 255.0) = 2.63, p = 0.050) and fell short of statistical significance for the Turkish sample (F(3, 146.0) = 2.65, p = 0.051) on weekdays.

Based on the overall sample adolescents with higher levels of depression scored medium to high on vertical individualism and spent more than fours a day on social media on weekdays, conversely those who scored low and hardly ever used social media had lower levels of depression (a difference of about 13 in total PHQ9 between a vertical individualism score of one and hardly ever using social media and a score of nine and using social media more than four hours every weekday). Higher depression levels were observed for adolescents who scored nine on vertical individualism and hardly ever used social media on weekdays (Total PHQ9 eight units higher than those who scored only one for vertical individualism). Adolescents who used social media 1–2 hours on a weekday had average depression levels that declined gradually as vertical individualism score increased.

A similar pattern of moderation was observed in the Turkish sample with a total PHQ9 difference of about 15 between a vertical individualism score of one and hardly ever using social media and a score of nine and using social media more than four hours every weekday. Higher depression levels were observed for adolescents who scored nine on vertical individualism and hardly ever used social media on weekdays (Total PHQ9 11 higher than those who scored only one for vertical individualism).

In the English Irish sample those with higher levels of depression scored medium to high on vertical individualism and spent more than four hours every weekday on social media. Those with the lowest level of depression scored high on vertical individualism and used social media for less than two hours per weekday (a difference of about 19 between this combination and a vertical individualism score of nine and using social media for more than four hours). It is worth noting that adolescents who scored low on vertical individualism and used social media for less than two hours in the English Irish sample also had raised levels of depression (a difference in 14 for Total PHQ9 between those scoring one and nine on vertical individualism). A similar pattern was observed for the English Irish sample at weekends.

For the overall sample depression levels varied in a similar way for horizontal individualism as was observed for vertical individualism. Those with higher levels of depression scored medium to high on horizontal individualism and used social media more than four hours every weekday. Those who scored one for horizontal individualism and hardly ever used social media had much lower levels of depression than those who scored nine (a difference of 10 in PHQ9 total).

Turkish adolescents who scored low to medium for horizontal individualism and hardly ever used social media had lower levels of depression than for all other combinations of time spent on social media during weekdays and horizontal individualism score. For these other combinations PHQ9 total varied by no more than five. The most protective combination was a horizontal individualism score of one and hardly ever using social media.

## Discussion

This study examined the association between time spent on social media and mental health outcomes in adolescents via the moderating effect of horizontal and vertical individualism-collectivism. A recent systematic review [1] that examined the influence of social media use on the mental health of adolescents informed the study aims of the present study. The systematic review findings revealed that the relationship between time spent on social media and mental health outcomes was inconsistent and complex. Few studies have examined the effect of other factors’ (e.g., gender, social support, rumination, sleep-related factors) on the observed relationship. There is dearth of literature on the effect of cultural differences on the use of social media and on the proposed association in adolescents. Using data from Türkiye, Ireland, and England, this is the first cross-sectional study that examined the moderating effect of vertical and horizontal dimensions of individualism and collectivism on the associations between the domains of social media (i.e., time spent weekdays and weekends) and mental health outcomes (i.e., anxiety, depression).

### Social media use and mental health outcomes

Findings from the cross-sectional study supported the hypothesis (H1a/b), that a significant association was found between the time spent on social media, especially spending more than four hours daily on school days and non-school days, and the symptoms of anxiety and depression in the entire sample. The findings reported here are consistent with some studies conducted with adolescents [52], but also contradict other study findings which has found no significant association between time spent on social media and mental health problems [7]. Despite mixed and inconsistent evidence on the relationship between screen time and mental health outcomes, there is considerable evidence in the literature showing that the increase in time spent on social media increases the risk of developing mental health problems in adolescents [52, 53]. Also, findings on the association between social media addiction and mental health problems in adolescents were consistent and showed that addiction to social media (i.e., uncontrollable urge to use social media) was positively associated with anxiety and depression [10, 54]. Therefore, a possible explanation for the observed associations in the current study is that adolescents who spend more than four hours daily on social media may be at heightened risk for being addicted to social media which may then lead to anxiety and depression.

Additional analyses on the split sample (Türkiye vs. England-Ireland) also showed that using social media more than four hours on weekdays was positively associated with depression and using social media more than two hours on weekend days was positively associated with anxiety among Turkish sample. The amount of time spent on social media needed to exceed four hours on weekdays to predict depression and exceed 2 hours on weekend days to predict anxiety in the Turkish sample. For the samples from England and Ireland, spending more than four hours on weekend days only was positively associated with both anxiety and depression. All these findings highlight that as time spent increases the risk of developing anxiety and depression increases. A significant association was not observed for weekdays in the England-Ireland sample.

As data from England and Ireland were collected during the Covid-19 pandemic, one possible explanation for the null finding for weekdays is that social media might have had protective effects by helping adolescents to stay virtually connected with their friends, entertain themselves, and cope with the negative impacts of the pandemic. A recent systematic literature review and meta-analysis revealed that social media platforms that facilitated access to connection, communication, and entertainment mitigated feelings of loneliness and stress in adolescents during the Covid-19 pandemic [55]. Another possible explanation for the null finding is that parental control over time spent on social media during weekdays (school days) may have acted as a protective factor for developing mental health problems [56].

Looking at the demographic information collected from each country, parental education was higher in the England-Ireland sample compared to the Turkish sample. One possible interpretation could be that educated parents are more aware of the possible effects of social media on their child’s mental health and so control over their child’s screen time or exposure to negative content on social media. On the other hand, in the England-Ireland sample, a significant association was observed between using social media more than four hours a day on weekend days and mental health outcomes (i.e., anxiety and depression). This can be explained by the increase in the perceived social isolation on weekend days during the Covid-19 pandemic. During the global pandemic, particularly in the initial stages, people were instructed by their governments to stay home, they were not allowed to go out, meet in person and socialize. On weekdays, adolescents might have been busy with study and schoolwork, however, during weekends, they may have had has less to do, which increased feelings of boredom and socially isolation. This may then have led to poor mental health outcomes. In contrast, spending more than four hours on social media during weekends may have had a positive influence on anxiety and depression in the English Irish adolescents. As discussed earlier, adolescents may have felt socially isolated especially during weekends when they had nothing to do, but social media may have helped them to cope with the negative impact of the pandemic and satisfy their needs for connection, communication, information, and entertainment, which may then have positively influenced their mental health.

### Individualism-collectivism, social media use and mental health outcomes

The findings indicate that there were no statistically significant interactions between the four cultural dimensions (INDCOL) and anxiety in relation to time spent on social media. However, significant interactions were observed for depression, particularly between vertical individualism and social media usage. This suggests that adolescents with higher levels of vertical individualism may be more susceptible to the negative effects of excessive social media use on depression, particularly on weekdays. These interactions were consistent across the overall sample and the Turkish and English-Irish subgroups, with the most notable effects observed in the English-Irish sample, both on weekdays and weekends. This highlights the potential moderating role of cultural values, especially individualism, in shaping the relationship between social media use and mental health outcomes like depression.

Vertical individualism, characterized by a focus on personal achievement, competition, and individual success, appears to exacerbate the depressive effects of excessive social media use. Adolescents with higher levels of vertical individualism may be more prone to experiencing negative emotional outcomes, such as depression, due to the heightened pressure to present an idealized version of themselves online and the increased reliance on social media for validation. This dynamic likely intensifies feelings of inadequacy and depressive symptoms when real-life experiences do not align with the curated personas they project online.

Furthermore, the moderating effect of horizontal individualism also warrants attention. Horizontal individualism, which values autonomy, independence, and self-expression without the emphasis on hierarchical structures, was found to interact significantly with social media use in relation to depression, albeit to a slightly lesser degree than vertical individualism. This suggests that while adolescents with higher horizontal individualism may experience increased depression linked to social media use, the effect is likely driven by the broader desire for self-expression and independence rather than social comparison and status attainment. For adolescents high in horizontal individualism, the emotional toll of social media may stem from the pressure to conform to individualized ideals, rather than the competition for social status inherent in vertical individualism.

Interestingly, amongst the Turkish sample, those who scored low to medium on horizontal individualism and hardly ever used social media experienced notably lower levels of depression. This suggests that, in the Turkish context, minimal social media engagement combined with lower levels of horizontal individualism may offer protective benefits against depression. This finding suggests that the most protective combination for Turkish adolescents was low horizontal individualism coupled with minimal social media use, which may be reflective of cultural values that prioritize communal or family-oriented engagement over digital self-expression.

### Strengths and limitations

The present study has several strengths. First, the study focused on mid-adolescence. During mid-adolescence, adolescents are in the stage of questioning and developing their personal and sexual identity [57]; they rely on their peers rather than their parents to affirm their identity by comparing themselves and their life with others [33]. As they spend time on social media, they may struggle with their ‘real self’ and, they may become more prone to the negative effects of social media. Second, previous studies have already explored the influence of other factors such as age [6, 8], gender [5], social support [58], and rumination [10] in attempt to explain the complexity in the relationship between social media use and mental health outcomes. The results of the present study contributed to the existing knowledge by providing evidence on the moderating effect of individualism and collectivism on the proposed relationship, which, to our best knowledge, has not been explored in adolescents before.

Several limitations, however, should be considered when interpreting the findings reported in the present study. First, this is a cross-sectional study, which cannot establish a cause-and-effect relationship between the exposure and outcome variables. Future studies should examine whether spent time on social media causes mental health problems or having mental health problems causes the increased time spent on social media through experimental studies. In addition to the linear causality, it would also be worth exploring a circular causality between time spent on social media and mental health outcomes which means that spending more time on social media may influence mental health and having mental health problems may then increase the time spent on social media. Second, the generalizability of the present results is limited to adolescents aged 14–16 years. This means results may differ in early adolescents, late adolescents, young adults, and adults. Third, data were collected from Türkiye in January 2020 before the Covid-19 outbreak, and then from England and Ireland during the outbreak. Although schools and samples in Türkiye were selected randomly, convenience and network sampling were used to select schools in England and Ireland due to the difficulty in accessing schools during the Covid-19 outbreak. Using convenience sampling for the England and Ireland sample may raise issues regarding generalizability of the present results and the increased risk of biased results (why some schools/parents/adolescents chose to take part and others not).

The discrepancies in smartphone ownership and internet access between Turkish participants and their British and Irish counterparts present significant implications for the study’s findings regarding social media’s influence on adolescent mental health. Although 48.9% of Turkish participants reported owning smartphones, the results revealed that a substantial 81.2% used smartphones to access social media, suggesting a reliance on devices owned by others. This dependence may restrict their engagement patterns and frequency of use, resulting in distinct social media experiences compared to the over 90% ownership reported among British and Irish participants, who likely engage with social media more independently and frequently. Such differences could shape varying mental health outcomes, as the Turkish participants’ higher rates of rare social media use (24–29%) may stem not only from personal preference but also from limitations imposed by shared device access.

The differing data collection methods—in-person surveys in Türkiye versus online surveys in England and Ireland—further complicate the interpretation of results. The higher internet penetration rates in England (95.3%) and Ireland (93.3%) [59, 60] suggest robust access to online resources, facilitating active social media engagement, whereas Türkiye’s lower rates (73.3% in 2020 and 77.2% in 2021) [61] may indicate inconsistent access for some individuals. While the differences in internet access could introduce potential biases, the strong connectivity in England and Ireland mitigates this concern for the online samples, which are likely to represent a digitally engaged demographic. These factors underscore the necessity of contextualizing our findings, as the variations in smartphone ownership and internet access, coupled with the differing data collection approaches, may skew the results and limit the generalizability of conclusions about social media’s impact on adolescent mental health across different cultural settings. Addressing these limitations is essential for a more nuanced understanding of the relationship between social media use and mental health in diverse populations.

In addition, using online surveys have several disadvantages besides its advantages such as time and effort saving, cost effectiveness, and flexibility. For example, although it is unlikely that adolescents had no internet access (remote learning due to Covid-19 pandemic), having no or limited access to internet may have prevented some from participating in the study. Although the survey completion rate was good (higher than 50%; 56.5% for the Ireland sample and 79% for the England sample), we believe the response rate in the England and Ireland sample was low considering the number of schools participating in the study. This could have led to non-response bias. The response rate might have been affected by several factors such as the email checking habits of both parents and adolescents, parents’ and adolescents’ attitudes toward research/surveys, level of interest, the lack of incentives/rewards, insufficient reminders, and the time they received the survey (whether it was a busy day/week/term, or exam week etc.). Therefore, this may have led differences between respondents and non-respondents regarding sample characteristics (e.g., age, gender, parents’ educational attainments, occupation etc.).

Moreover, the use of self-reported measures has limitations. Participants reported the length of their membership with social media, the number of followers and the number of hours they spend on social media on school days and non-school days. They may have under or over-estimated these amounts due to issues of recall, which again may lead to bias. Recall bias may strengthen or weaken the observed associations. For example, when adolescents recall less than actual amounts of time they spend on social media, associations may suggest that less time spent on social media is associated with mental health problems. What is more, the relationships between the time spent on social media and mental health outcomes might be affected by other factors that were not controlled for in the current study. For example, we did not collect information about respondents’ online activities, their passive and active use of social media. Additional measures that assess participants’ social media-related activities would have been informative. Thus, the present findings should be interpreted with caution. Future studies should use more comprehensive measures to evaluate participants’ online activities, their passive and active use of social media, and see if the present study’s findings can be replicated or not.

### Implications and recommendations

The findings of the present study have several implications. First, the findings add to the existing evidence regarding the association between time spent on social media and mental health outcomes in adolescents. Results suggest that time spent, especially more than four hours daily, needs increased attention in terms of being an indicator of mental health in adolescents. As the amount of time spent on social media may be overestimated or underestimated, this also has implications for the present study. Future studies may use different approaches (e.g., checking or asking participants to check time spent on social media through the phone/apps) instead of self-report questions for more reliable answers to the amount of time spent. Also, the influence of social media on the mental health of adolescents may differ from one person to another. Qualitative studies may help improve our understanding of the nature of this complex relationship and give important insight into the underlying factors (i.e., protective vs risk factors) that may explain the association between the two. Therefore, more qualitative research is needed to explore the influence of social media on the mental health of adolescents from adolescents’ own perspectives and lived experiences.

Regarding the moderating effects of cultural dimensions, we found that vertical individualism moderated the relationship between time spent on social media and depression across the entire sample, as well as in the Turkish and English Irish subgroups. Additionally, horizontal individualism moderated this association in both the overall sample and the Turkish sample. These findings underscore the importance of considering cultural context, particularly individualistic cultural orientations, when designing interventions to mitigate the negative mental health effects of social media. It is essential that interventions not only address the amount of time adolescents spend on social media but also incorporate an understanding of the cultural values that shape how they perceive and engage with online platforms. Specifically, the role of cultural dimensions such as vertical and horizontal individualism should be explored further, as they appear to moderate the relationship between social media use and mental health outcomes. For instance, future research could investigate whether the association between time spent on social media and mental health is stronger or weaker for adolescents with higher levels of vertical individualism. A deeper exploration of these cultural factors will allow for more tailored, culturally sensitive interventions that consider the unique pressures faced by adolescents in different cultural contexts.

Finally, the present study findings revealed that the prevalence of moderate to severe anxiety and depression were about 42% and 44% respectively in the entire sample, which was a concerning finding. As discussed earlier, adolescence is a critical and vulnerable period for the development of mental health problems, especially anxiety and depression. In most cases, adolescents with mental health problems are not detected and remain untreated and thus their experience of mental health problems may persist into adulthood or even result in destructive consequences during adolescence or adulthood (e.g., self-harm) [62–64]. Adolescents with mental health problems experience several challenges such as social exclusion, stigma that prevent adolescents from seeking help, educational difficulties, risk-taking behaviours such as self-harm and substance use, physical ill-health, and violation of human rights [64]. The high prevalence of anxiety and depression revealed in this school-based survey highlights the importance of school-based screening and detecting adolescents at risk of mental health problems and risk-taking behaviour. School-based screening and intervention are particularly important for those who will otherwise not seek professional help due to stigma, a lack of knowledge, ignorance, low socio-economic status, and cultural and parental factors.

## Conclusions

This study highlights that spending more time on social media, especially more than four hours daily, was significantly associated with increased anxiety and depression levels. Horizontal and vertical individualism moderated the association between time spent on social media and depression for the overall and Türkiye sample. In English Irish sample time spent on social media was moderated just by vertical individualism. How time spent on social media impacts mental health outcomes in adolescents was advanced in this study by providing supporting and contrasting evidence to the existing knowledge as well as new evidence on the moderating effect of cultural dimensions on the observed associations. Findings highlight the need for further studies to examine the direction of the observed relationship. Also, more qualitative research is needed to better understand the mental health implications of social media from the perspectives of adolescents.

## Supporting information

S1 Raw data (SAV)
