# Correction: Moderating effect of cultural differences on the association between social media use and mental health outcomes in adolescents: A cross-cultural comparison study

**DOI:** 10.1371/journal.pone.0330649

**Published:** 2025-08-20

**Authors:** Betul Keles-Gordesli, Mary Leamy, Trevor Murrells, Annmarie Grealish

The first author’s name is spelled incorrectly. The correct name is: Betul Keles-Gordesli. The correct citation is: Keles- Gordesli B, Leamy M, Murrells T, Grealish A (2024) Moderating effect of cultural differences on the association between social media use and mental health outcomes in adolescents: A cross-cultural comparison study. PLoS ONE 19(12): e0316365. https://doi.org/10.1371/journal.pone.0316365.
